# Finding the Right Balance: Challenges in Optimising the Promise of Complexity Research for NCD Best-Buys Implementation and Adoption: Comment on "Barriers and Opportunities for WHO ‘Best Buys’ Non-communicable Disease Policy Adoption and Implementation From a Political Economy Perspective: A Complexity Systematic Review"

**DOI:** 10.34172/ijhpm.9040

**Published:** 2025-07-09

**Authors:** Pragati B. Hebbar, Upendra M. Bhojani, Prashanth Nuggehalli Srinivas

**Affiliations:** ^1^Center for Commercial Determinant of Health, Institute of Public Health Bengaluru, Bengaluru, India.; ^2^Centre for Health Systems, Institute of Public Health Bengaluru, Bengaluru, India.

**Keywords:** Non-communicable Diseases, Political Economy Analysis, Realist Methods, Complexity Research, Best Buys

## Abstract

There is a growing interest in complexity research. A recent systematic review by Loffreda et al attempted to study the barriers and opportunities for the adoption and implementation of the "best buys" for non-communicable diseases (NCDs) from a political economy perspective. In this commentary we take forward the discussion on the NCD best-buys by comparing the findings of the article with one of the risk factors of tobacco use and its control in India. We reflect on the challenges in actualizing the promise of research methods and approaches while studying such complex interventions like the NCD best buys. The balance of studying complexity while still keeping the findings translatable at country levels. Future research could potentially use a comparative lens focusing on either industry/government or actor behaviour across the different risk factors to facilitate cross learning, anticipate and pre-empt adverse policy decisions and implementation outcomes.

## Engaging With Complexity

 There is a growing interest in complexity and studying complex health interventions with a range of guidance and tools to help engage with complexity.^1,2^ In this backdrop we welcome the contribution by Loffreda et al^3^ in explaining adoption and implementation of policies aimed at preventing and managing non-communicable diseases (NCDs). We appreciate the authors’ ambitious attempt at studying many NCD policies together. This builds complexity in research, and is desirable given the huge potential for cross-learnings for policy adoption/implementation. Authors’ choice to focus on the “best buys” being promoted globally through the World Health Organization (WHO) and other mechanisms keeps the findings strategically relevant to these global actors and national governments. The best buys encompass six objectives, nine voluntary targets and 25 indicators which has recently been expanded to 28 and three strategic directions for the implementation roadmap 2023-2030.^4^ We are especially encouraged with authors’ emphasis in recognising the policy adoption and implementation as complex interventions rather than looking at them as techno-managerial processes, and hence, the centrality of political economy approach in studying these phenomena. Accordingly, the authors use a mix of methods (and approaches) such as realist evaluation, political economy analysis, complexity science and others. While the topic under study of NCD is highly relevant and the focus on implementation and complexity has been broached we identify issues that could benefit from a wider discussion in the interest of scholarly dialogue and advancing scholarship in this domain. In this commentary, we extend the discussion on researching complexity in the context of NCD policies building on what we identify as some of the challenges in the paper. In doing so, we draw on insights we developed from similar work (researching complexity in tobacco control policy and implementation) in India.

## Challenges in Optimising the Methodological Promise

 One of the major challenges is to optimise the promise of methods in researching complexity. Does the adoption of a complexity lens in synthesising the findings from the review justify this term *“complexity systematic review,”* given that this is not a commonly used term.^1^ While the authors make a case for “best buys” to be complex interventions and the need to use political economy to study them the article does not define or explain why they consider this systematic review (intervention, method and/or findings?) complex. Loffreda et al^3^ describe at least seven methods and approaches such as complexity approach, realist review tools, causal loop diagrams, complexity assessment tool, three I’s political economy analysis, theory of agency, and power and qualitative thematic comparative analysis. While these are possibly the best we have for researching complexity, one keeps longing to see the promise of these methods actualised in the results/analysis section of the paper. For instance the paper mentions political economy analysis, but there is a lack of substantive information on how such analysis was conducted and how it informed the overall study findings. Similarly the actor and context focus raised in the methods section is not reflected in the results. We find that political analysis approach is indeed very useful in understanding tobacco-related policy adoption and implementation in India. However, in our experience, diverse types of literature beyond peer-reviewed scientific publications (including news media, editorials, civil society writings, budgets, parliamentary questions/debates, and litigations) is especially useful in capturing data needed for political economy analysis.^5-7^

 Further, while the realist review was carried, the choice to use a causal loop diagram (for initial program theory) and qualitative thematic comparative analysis (for data synthesis) seem to fall short of bringing out the central analytical heuristic of context-mechanism-outcome of realist methods. While it is not necessary to stick to traditional heuristic, it would be useful to learn authors’ reflections on how the tools they used helped them optimise the realist review principles.

 The authors rightly emphasised the importance of context and the need for the methods to consider contextual diversity/insights while engaging in complexity research. However, by design, the study focused at national/country level. The policy adoption and especially implementation requires considerations of sub-national jurisdictions and contexts, especially so in federal democracies. For example, in India, implementation of tobacco control policies as well as tobacco use prevalence and the rate of decline in tobacco use prevalence across time (as potential outcome of tobacco control policies) vary widely across states.^8^ We demonstrate how state (sub-national) level context (including state regimes, public policy related to tobacco, tobacco industry interference, and civil society action) has deterministic impact on tobacco use prevalence and can explain differential decline in tobacco use across Indian states over time. Furthermore, while it is interesting to see authors using a concept of “fragility” (a term routinely used in global security discourse, often driven by the Organisation for Economic Co-operation and Development countries) to characterise country contexts, the rationale for using it is not elaborated in the paper. We appreciate that the concept embodies a variety of interlinked contextual conditions. The concept of “fragility” itself is fluid – countries “could be fragile in its own way” to an extent that some argue futility of standardising fragility measures and comparisons. We need to focus more on how to categorise countries (national context) that goes beyond frequently used income-categories and use concepts that capture a range of relevant conditions that are proven or that can be theoretically linked to NCD policy adoption/implementation.^9^

 Engaging with complexity is crucial and there is a promise in methods the authors used, one may need to balance the degree of complexity to be studied with the methodological challenges. Maybe the paper took on too much, in terms of its geographical scope (global), a range of policies to be studied, both policy adoption and implementation phases to be studied. This seems to come at the cost of details one can engage with at country level and hence, limiting the potential to create differentiated explanations at country level. For example, the key findings related to reducing tobacco consumption seem generic highlighting the need for strong legal framework. While the WHO Framework Convention on Tobacco Control (FCTC) highlighted in the paper is indeed important, India – like many other countries – have variable degree of adoption and implementation of policies (including policies beyond FCTC). Nuanced sub-national level theories explain mechanisms of collective action, felt accountability, fear and prioritization of tobacco control in Indian states where the implementation outcomes of tobacco control policies vary widely.^10^ Further human resources having limited capacity and skills is not seen as a major barrier for tobacco control policy implementation which is in contrast to the findings in our recent realist review on how tobacco control policies work in low- and middle-income country settings.^11^ Training and capacity building amongst authorised implementation personnel leading to knowledge sharing and exchange is an important strategy to improve the readiness of the whole organization to implement tobacco control laws. In the main facilitators too the whole-of-society approach, surveillance system and local evidence and locally driven policies are not seen as facilitators for tobacco control. These again are quite contrary to our experience as policy-makers often ask for local data to substantiate their policy actions and do not rely on global data or data from other countries for their decisions. Hence strengthening surveillance systems and setting up and maintaining disease specific registries is crucial. For example, after making cancer a notifiable disease in Karnataka, one of the south Indian states, a cancer registry was set up, multiple states banned sale and production of chewing tobacco products taking actions at sub-national levels. Although such a nation-wide process of notification is not yet implemented several states have made cancer a notifiable disease.

 Beyond the key findings presented in the tables we examine the results in the narrative synthesis structured based on the three variables of the causal loop diagram. One of the findings based on articles from Kenya, Nigeria, the Pacific, and Cameroon states that countries that grow tobacco have limited effectiveness in both formulating and implementing tobacco control best buy interventions. India and Brazil are two of the top three producers of tobacco despite which India has been an early signatory of the WHO-FCTC in 2004 followed by Brazil in 2006 and both countries have enacted several provisions at the national and sub-national levels and witnessed reduction in tobacco use over the past two decades.^12^ Within India the state of Karnataka is one of the large growers of tobacco but has made substantial progress in terms of highest tobacco control law enforcement and implementation.^13^ These instances depict that although tobacco growing can be a barrier beyond this barrier there are several other factors at play that can shape the outcomes of policy processes necessitating studying policy implementation at sub-national levels. The paper rightly points out the need for low- and middle-income countries to focus on supporting tobacco growers to economically viable alternative options. Another important aspect that the paper draws our attention to is monitoring, evaluation and surveillance systems like the WHO STEPwise approach and the need to invest in these and sustain them. The Global Adult Tobacco Survey provides standardized data on consumption and proxy indicators of implementation but countries can focus on building capacity to collect local data and analyse it to understand trends.

## Way Forward

 Overall the paper broadens the scope to look at NCD policies, but the focus on “best buys” could still be limiting – this emphasis on four risk factors and related interventions (including emphasis on cost effectiveness) ignores many other important drivers of NCDs and the differentiated NCD epidemics across contexts/societies. Based on such an overarching analysis it appears that detailed studies of risk factors separately or comparatively might yield more policy relevant insights and depth in findings. Future research could potentially use a comparative lens focusing on either industry/government or actor behaviour across the different risk factors to facilitate cross learning, anticipate and pre-empt adverse policy decisions and implementation outcomes. With the aim being to strike a right balance of the degree of complexity being studied with the methodologic limitations to ultimately lead to a whole (explanation/theorization) that is greater than its parts.

## Ethical issues

 Not applicable.

## Conflicts of interest

 Authors declare that they have no conflicts of interest.
